# From Offline to Inline Without Pain: A Practical Framework for Translating Offline MR Reconstructions to Inline Deployment Using the Gadgetron Platform

**DOI:** 10.1002/mrm.70304

**Published:** 2026-04-01

**Authors:** Zihan Ning, Yannick Brackenier, Sarah McElroy, Sara Neves Silva, Lucilio Cordero‐Grande, Samuel Rot, Liane S. Canas, Rebecca E. Thornley, David Leitão, Davide Poccecai, Andrew Cantell, Rene Kerosi, Anthony N. Price, Jon Cleary, Donald J. Tournier, Jana Hutter, Philippa Bridgen, Pierluigi Di Cio, Michela Cleri, Inka Granlund, Lucy Billimoria, Yasmin Blunck, Shaihan Malik, Marc Modat, Sebastien Ourselin, Claire J. Steves, Joseph V. Hajnal

**Affiliations:** ^1^ Imaging Physics and Engineering Research Department, School of Biomedical Engineering and Imaging Sciences Kings College London London UK; ^2^ MR Research Collaborations Siemens Healthcare Limited Camberley UK; ^3^ Biomedical Image Technologies, ETSI Telecomunicación Universidad Politécnica de Madrid & CIBER‐BBN, ISCIII Madrid Spain; ^4^ NMR Unit, Queen Square MS Centre, Queen Square Institute of Neurology University College London London UK; ^5^ Guy's and St Thomas' NHS Foundation Trust London UK; ^6^ London Collaborative Ultra High Field System (LoCUS) Kings College London London UK; ^7^ Department of Biomedical Engineering The University of Melbourne Parkville Australia; ^8^ School of Biomedical Engineering and Imaging Sciences King's College London London UK; ^9^ Department of Twin Research and Genetic Epidemiology King's College London London UK

**Keywords:** inline MR reconstruction, MR translation, open platform for reconstruction

## Abstract

**Purpose:**

To develop and validate a practical, open‐source framework to overcome common issues in inline deployment of established offline MR reconstruction, including (1) scan disruption from lengthy reconstructions, (2) limited support for multi‐scan input reconstructions, (3) needs to adapt scripts for different raw‐data formats, and (4) limited guidance and experience in retaining scanner reconstructions and applying scanner‐based post‐processing to custom‐reconstructed images.

**Methods:**

The framework builds upon the Gadgetron platform as implemented on Siemens scanners and includes: (1) a general input converter to convert Gadgetron‐used ISMRMRD format raw into a Siemens format raw structure, facilitating reuse of code; (2) an asynchronous trigger‐and‐retrieve mechanism enabling long custom reconstructions without delaying scanner processes; (3) resource‐aware scheduling for parallel execution of reconstructions; (4) integrated file management to support multi‐scan inputs; and (5) preservation of scanner‐based reconstructions and post‐processing. The framework was validated on 2 Siemens scanners for SENSE, AlignedSENSE, and NUFFT reconstructions, and in a large‐cohort study.

**Results:**

Minimum code modification for inline deployment was demonstrated, and all reconstructions were successfully executed inline without disrupting scanner workflows. Images were retrieved automatically via retrieval scans or manually via retro‐reconstruction, with scanner‐based post‐processing applied to custom outputs. Multi‐sequence reconstructions were executed using GPU‐aware scheduling, confirming feasibility for large‐scale applications. In 480 examinations, inline reconstructions were retrieved in 99% of cases without disruptions.

**Conclusion:**

The framework lowers the technical barrier to inline deployment of offline reconstructions, enabling robust, scalable, and post‐processing‐compatible integration. It is openly available with documentation and demonstration cases to support reproducibility and community adoption.

## 1 Introduction

Advanced MR reconstruction and post‐processing techniques are progressing rapidly, but adoption in clinical settings and large‐scale research remains limited due to challenges in integration with normal scanner workflows. Without this inline integration, executing custom algorithms demands considerable effort. Extraction of raw (k‐space) data and transmission to a suitable compute resource is generally manual, return of outputs to the scanner database is often infeasible and import to a suitable PACS (Picture Archiving and Communication System) can be cumbersome. Offline processes may not allow for standard post‐processing (e.g., bias field and distortion correction), limiting compatibility with other acquired images.

Hitherto, integration of custom reconstructions into seamless workflows has required researchers to work within proprietary native scanner frameworks. Although feasible under suitable research agreements, it has required specialist knowledge and often considerable reworking of code. There are open‐source platforms, such as Yarra [1] and Gadgetron [2, 3], designed for prototyping and integration of reconstructions into workflows, and these have the advantage of supporting widely used high‐level languages like Python and MATLAB, reducing reimplementation effort. Yarra [1] is a semi‐inline solution that automates raw data collection, custom reconstruction triggering, and PACS or workstations export, though it lacks inline image review capabilities. In contrast, Gadgetron provides both modular reconstruction/processing components (i.e., “Gadgets”) and inline data‐streaming functionality (e.g., the IceGadgetron program on Siemens platforms) that enables the launch of custom reconstructions and return of resulting images directly to the scanner console [2, 3]. More recently, vendors have begun embracing open‐source toolboxes (e.g., Gadgetron and BART) by offering hybrid solutions integrated into their proprietary workflows [4], such as FIRE [5] and Open Recon [6] for Siemens, GyroTools for Philips [7], and Orchestra Live for GE [8].

Moving from the platforms to data formats, whereas DICOM provides a universal interchange format for medical images, there has historically been no equivalent for k‐space data. ISMRMRD format [9] provides a step towards open standards, is native for Gadgetron and has been adopted by some vendors for their next generation frameworks (e.g., Siemens [10] FIRE and Open Recon, GE [11] Orchestra, and Philips [12] Recon2.0). However, many existing reconstruction algorithms have been written for proprietary raw data formats, which limits their immediate compatibility with ISMRMRD.

There is a growing range of available options for creating inline reconstruction workflows, but many practical challenges remain. Common issues include: (1) scanning disruption or delay if inline reconstructions are time‐consuming; (2) limited support for reconstructions requiring multiple inputs from different scans; (3) the need to adopt different raw data formats; (4) limited guidance and experience in retaining scanner reconstructions for early review or comparison, and in applying scanner‐based post‐processing to custom‐reconstructed images.

To address these challenges, we developed a practical open‐source framework based on Gadgetron. The aim is to enable workflow‐compatible inline deployment of established offline MR reconstructions, regardless of computation time or the number/source of inputs, while requiring minimal adaptation to the original scripts. Full inline operation places custom‐reconstructed images directly into the native scanner database, which is ideal for integration with established workflows and standard data management pathways (e.g., connection to PACS). There are governance requirements associated with such complete integration, which is critical to acknowledge and address these appropriately, but how that is done is beyond the scope of this paper.

The proposed framework, which is implemented for Siemens scanners, incorporates the following key features:

- *Maximum Script Reuse for Rapid Prototyping*: ISMRMRD‐formatted data is converted into a Siemens Twix‐like structure, so that original offline code could be maximally reused.
- *Parallel and Non‐Disruptive Execution*: Supports an asynchronous trigger‐and‐retrieval mechanism [13] to avoid delays in subsequent scans and reconstructions, and uses multi‐GPU scheduling with resource monitoring to run custom reconstructions efficiently in parallel.
- *Integrated Multi‐input Data Management*: Supports structured scan‐origin tracking for all data files to enable input readiness checks for multi‐input reconstructions, and ensuring reliable automatic image retrieval for all reconstructions.
- *Preservation of Scanner Reconstruction and Post‐processing*: Enables early image review through retained scanner reconstructions, and supports scanner‐based post‐processing on custom outputs to ensure consistency.
- *Robust Operation*: Real‐time logging and error tracking ensure resilience and recovery without requiring rescans or interrupting acquisitions.

This framework lowers technical barriers for translating offline methods into inline workflows, enabling efficient and scalable deployment in clinical protocols and large‐cohort studies. Validation was performed on three aspects: (1) feasibility for inline implementation of established offline reconstructions, (2) feasibility for multi‐sequence implementation within one examination, and (3) robustness.

## 2 Methods

### 2.1 Physical Infrastructure and Framework Architecture

**FIGURE 1:**
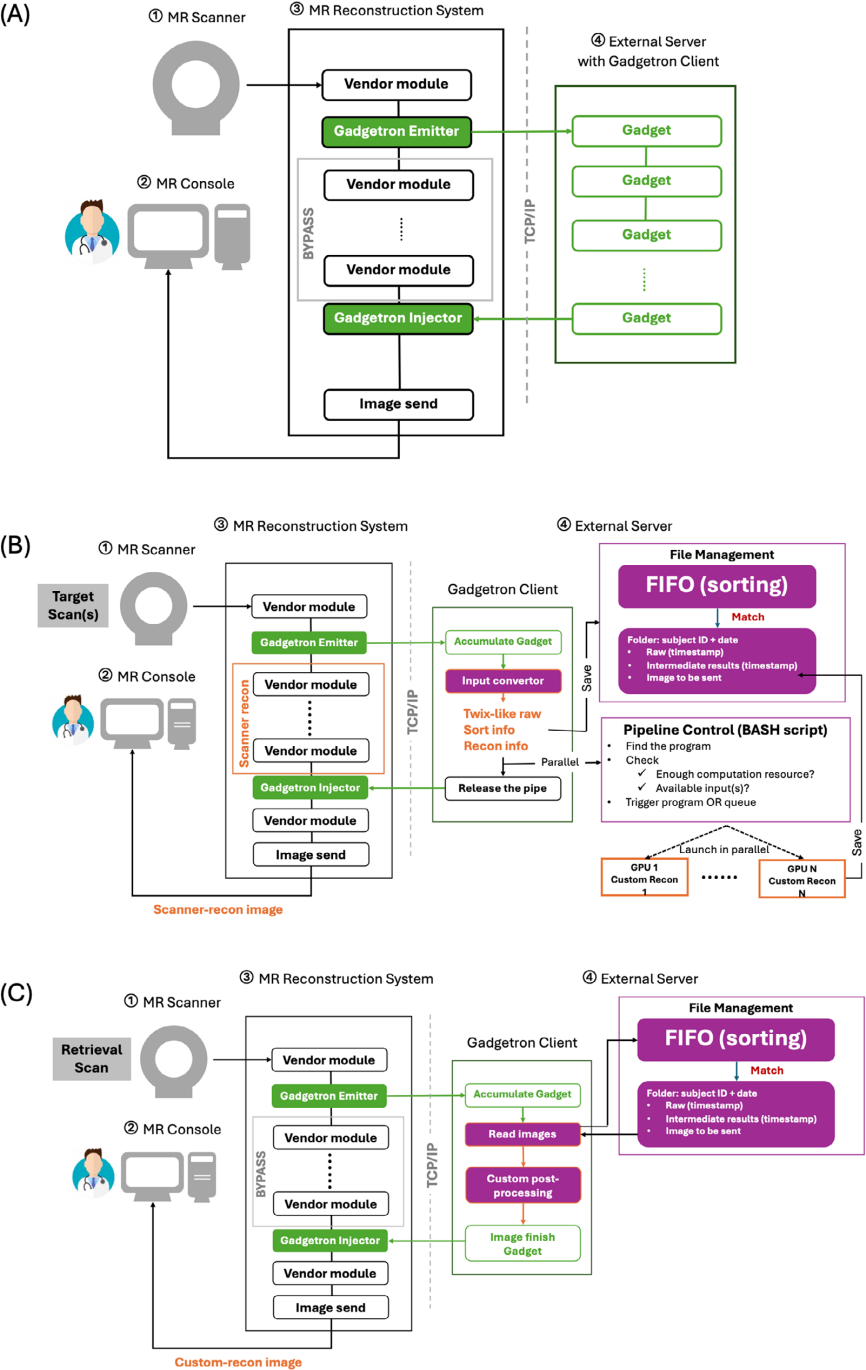
The diagram illustrates the conventional Gadgetron workflow (A) and the workflow with the proposed framework, including the target scan (B) and retrieval scan/retro‐reconstruction (C). The proposed framework was built upon four core components: The MR scanner, the MR console, the MR reconstruction system with the IceGadgetron program (enabling the emitter and injector modules), and an external reconstruction server with the Gadgetron client. For the target scan, the framework triggers a parallel pathway for raw data conversion, saving, and reconstruction, while preserving the conventional scanner reconstruction for early review on the scanner console. Once custom reconstructions complete on the external server, images can be retrieved to the console via retrieval scans or retro‐reconstructions, with scanner post‐processing applied.

The proposed framework operates on a physical infrastructure comprising four core components (Figure 1A): (1) the MR scanner (magnet, gradient and RF systems), (2) the MR console, (3) the MR reconstruction system with the IceGadgetron program installed enabling related modules, and (4) an external reconstruction server with the Gadgetron client [3].

#### 2.1.1 Standard IceGadgetron Pipeline

This study uses the implementation of Gadgetron that operates on Siemens scanners, known as IceGadgetron. It provides functionality for inline data‐streaming, which integrates with the native scanner reconstruction pipeline using an “emitter” module to send raw data to the external server and an “injector” module to return images. After acquisition, the emitter converts vendor‐specific raw data into ISMRMRD format [9], using predefined markup documents called ParameterMaps [10], and transmits it to the server via TCP/IP connection. Intermediate scanner reconstruction modules are bypassed, thus no scanner‐reconstructed images are generated. Instead, on the external server, a customized chain of Gadgetron programs, named “Gadgets,” performs pre‐processing and user‐defined reconstruction, with support for Python and MATLAB implementations. After processing, final images are returned to the scanner reconstruction workflow at the injector. The images are stored in the scanner database, and made available for console review or export (e.g., to PACS). Since the scanner workflow waits for image return and operates sequentially, long reconstructions can delay subsequent tasks. Gadgetron provides a built‐in storage server [14] for persisting and reusing data from previous scans. While it offers powerful native capabilities, extending it to support reconstructions that rely on heterogeneous inputs, flexible loading by third‐party programs, or inputs that are not yet available when a target scan is triggered would require substantial development of custom Gadgets and coordination logic.

#### 2.1.2 Rapid Prototyping With the Framework

Most reconstruction methods are first developed offline using vendor‐specific raw data, which are generally incompatible with ISMRMRD during inline translation. On Siemens platforms, the ISMRMRD output from the Gadgetron emitter differs significantly from the original “Twix” format in both header structure and k‐space organization. Furthermore, only limited headers are converted by default, and standard preprocessing by default Gadgets (e.g., noise prewhitening, gridding) are required for data collection but may alter data or consume dependent lines (e.g., noise scans), further increasing mismatches. Consequently, existing reconstruction scripts often require extensive modification for inline deployment.

To address these issues, the proposed framework omits default Gadgetron preprocessing steps, deploying only the Accumulate Gadget to collect raw k‐space lines (Figure 1B). An updated ParameterMap ensures that key Twix headers are preserved in the ISMRMRD output. A general input converter is then provided and applied to reconstruct a Twix‐like structure. Compared to some similar converters (e.g., fire_mapVBVD [15]), it not only reorganizes the k‐space lines into a matrix format consistent with outputs from mapVBVD [16] (i.e., the most wide‐using Siemens raw reader), but also restores the expected header structure and populates key header fields for reconstructions and processing.

#### 2.1.3 Multi‐Input Processing and Asynchronous Operation

To support long and/or multi‐input reconstructions, the framework employs an asynchronous trigger‐and‐retrieval mechanism [13] with parallel processing and integrated data management (Figure 1B,C). Each reconstruction involves a “target scan” to acquire data and trigger reconstruction, and a “retrieval scan” to return images back to the console. Alternatively, Siemens' retro‐reconstruction feature can be used for image retrieval after the examination session.

During the target scan, k‐space data are streamed to the external server via the emitter module. A Read&Save handler receives the ISMRMRD‐formatted streaming data, applies the general input converter to reconstruct a Twix‐like structure, and saves it (currently in mat format file) to an organized folder named with subject ID and date in the external server. Files are tagged with category (raw data or image to retrieve), sequence name, and timestamp to prevent overwriting. After saving, the handler exits and triggers a control script, while the Gadgetron workflow completes without returning images to the injector, allowing the scanner reconstruction pipeline to proceed without delay. If immediate image feedback is required and native reconstruction is feasible, the emitter can retain intermediate modules, allowing parallel scanner‐based reconstruction.

The control script then checks two conditions before launching the reconstruction: (1) availability of all required inputs and (2) sufficient computational resources (e.g., GPU). If both are met, the custom reconstruction is launched; otherwise, the process is automatically queued until conditions satisfied. Two log files are maintained: a resource log for tracking resource usage, and a computation log for monitoring progress and errors.

For image retrieval, either a retrieval scan (i.e., a short dummy scan, < 5 s) or retro‐reconstruction can be used. In both cases, the emitter sends a matrix‐size‐aligned raw to the external server but discarded; instead, saved images are located, loaded, and returned via the injector to the MR reconstruction workflow (Figure 1C). The injector precedes scanner post‐processing modules to ensure consistency with native images. Retrieval sequences are short sequences, which match the target sequence in matrix size to ensure successful image return, and are linked to the scanner pipeline configured for retrieval rather than for triggering custom reconstructions. Once set up, retrieval scans enable automatic image return within the protocol, whereas retro‐reconstruction requires no new sequence setup but manual pipeline selection on the MR console interface. For users without Siemens sequence programming experience, the simplest way is to use retro‐reconstruction for retrieval, or to create a retrieval scan by duplicating the target scan and switching the IceGadgetron configuration from reconstruction triggering to retrieval. The sequence can be modified to reduce its “scanning” time, but it is important to preserve the original reconstruction matrix. Retrieval scans generate image headers from their own sequence settings, so header mismatches with the target scan may occur, while retro‐reconstruction inherently preserves alignment. For retrieval scans in our framework, target sequences were duplicated and programed to acquire only a single central k‐space line when the retrieval pipeline was selected from a custom‐programed drop‐down menu, ensuring aligned headers and rapid retrieval (see Supporting Information A for detailed comparisons). Each target scan requires one retrieval scan or once retro‐reconstruction for image retrieval.

### 2.2 Framework Validation

#### 2.2.1 Feasibility for Inline Implementation of Established Offline Reconstructions

Three pre‐existing reconstruction algorithms have been implemented inline via the proposed framework [17]: (1) conventional SENSE with linear Cartesian trajectory [18]; (2) AlignedSENSE [19] with the DISORDER trajectory [20] for motion correction reconstruction using a rigid body motion model; and (3) NUFFT [21] with radial trajectory data for ^22^Na imaging [22, 23]. For SENSE and AlignedSENSE reconstruction, coil sensitivity estimation was performed by ESPIRiT [24] with options for embedded auto‐calibration lines or external reference scans (the latter used to demonstrate multi‐input reconstructions). All three reconstructions were developed offline and integrated inline using MATLAB scripts. The detailed information about scanner, external servers and sequence parameters are provided in Tables S1 and S2.

#### 2.2.2 Feasibility of Multiple Inline‐Reconstructions Within One Examination

To evaluate the framework's capabilities to achieve multiple asynchronous inline reconstructions within a single examination, a healthy volunteer was imaged with the protocol: (1) an external reference scan for ESPIRiT‐based coil sensitivity estimation; (2) T1‐MPRAGE, (3) FLAIR, (4) SWI, and (5) T2W SPACE, all with inline implemented AlignedSENSE reconstruction (detailed parameters in Table S3). FLAIR and T2W SPACE used coil sensitivity maps from the external reference, while the others relied on embedded auto‐calibration lines.

#### 2.2.3 Robustness

Framework robustness was validated in a large‐scale cohort study, the TwinsUK Project [25, 26], incorporating a motion‐corrected T1‐MPRAGE sequence with DISORDER trajectory and inline AlignedSENSE reconstruction. All scans acquired between April 3, 2024 and April 4, 2025 were reviewed to assess: (1) any disruption to scans or reconstructions, and (2) successful retrieval of both scanner‐ and AlignedSENSE‐reconstructed images.

## 3 Results

### 3.1 Feasibility for Inline Implementation of Established Offline Reconstruction Scripts

The proposed framework is open‐sourced along with the three demonstration cases, including both the original offline code and their inline versions, accompanied by a detailed user manual [17]: https://github.com/ZihanNing/Practical_Inline_Recon_Framework‐public.

**FIGURE 2:**
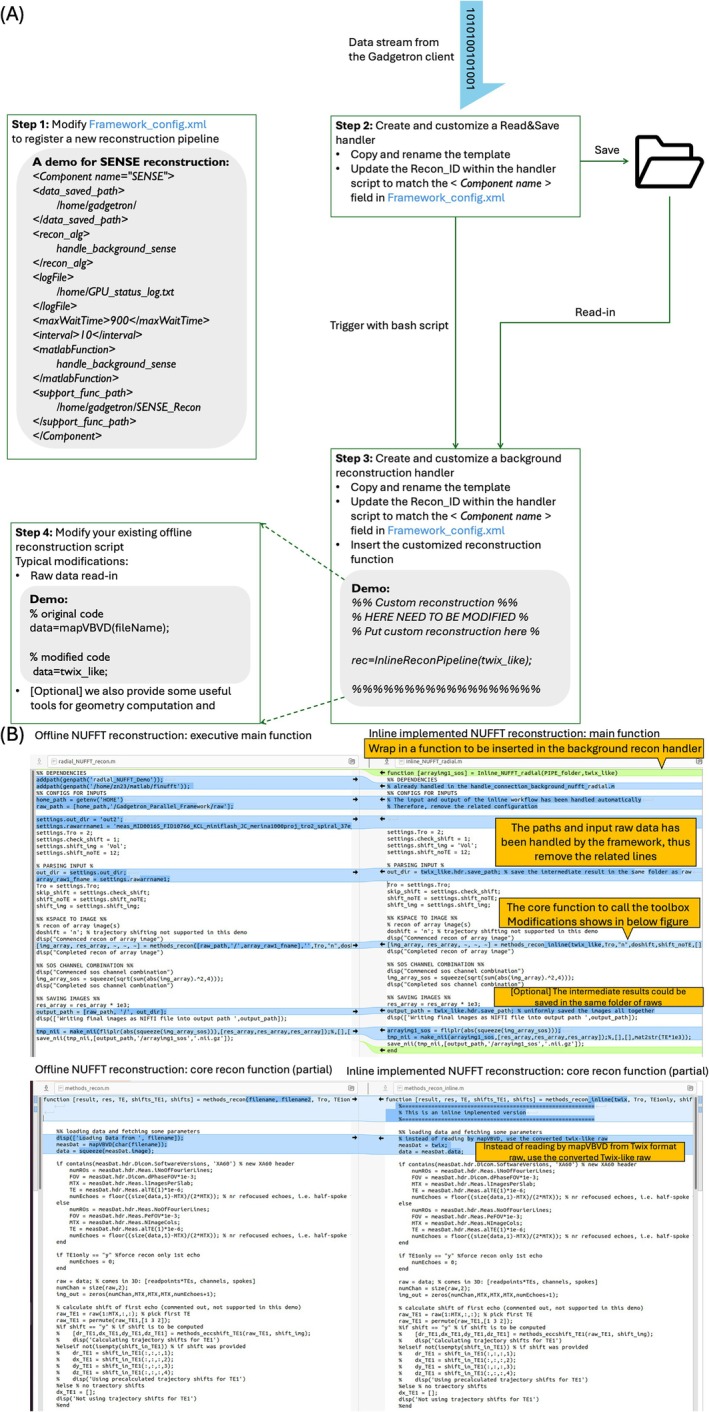
A diagram to demonstrate the code‐level modifications (in 4 steps) to translate an offline reconstruction for inline use. Essential modifications include: (1) modify the centralized configuration file (Framework_config.xml) to register a new pipeline (i.e., defining “Recon_ID” and relative configurations); (2) define a Read&Save handler by copying the provided template and specifying the matching “Recon_ID”; (3) define a background reconstruction handler similarly; (4) adapt the existing offline reconstruction script and wrap it as a function to insert in the background reconstruction handler. Since the ISMRMRD format raw has been converted back to a twix‐like structure, the existing offline reconstruction script could be maximally reused.

To illustrate the ease of translation, Figure 2A outlines the 4‐step process for adapting a Twix‐input offline reconstruction for inline use: (1) registering a new pipeline in the centralized configuration file, defining (2) Read&Save and (3) background reconstruction handlers from the templates, and (4) wrapping and adapting the original script to be inserted in the background reconstruction handler. Since the ISMRMRD format raw has been converted back to a Twix‐like structure, the original script could be largely reused. For minimal changes, replace vendor‐specific data reading functions with access to the provided Twix‐like input. For the three demonstrations, minimal modifications (% of code lines changed) required for inline implementation were: 0.07% (18/26,693) for NUFFT, 0.19% (65/33 972) for SENSE, and 0.04% (47/106 522) for AlignedSENSE. Exact code‐level differences are available in the repository [17]. In the open‐sourced SENSE and AlignedSENSE versions, additional changes were made to support external reference scans (i.e., 0.84%, 284/33 972 for SENSE; 0.40%, 428/106 522 for AlignedSENSE).

**FIGURE 3:**
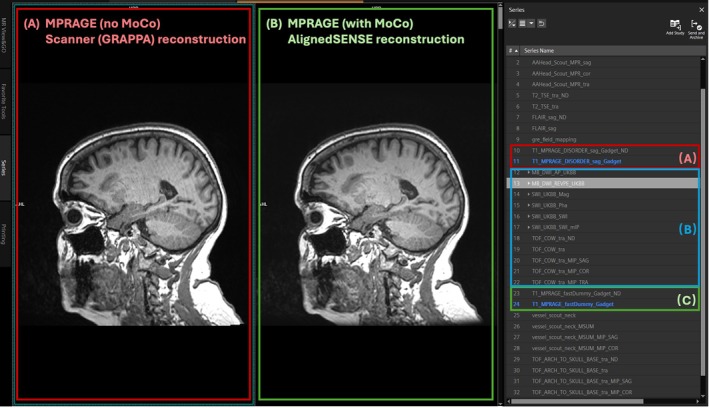
Screenshots from the MR console illustrate a protocol in which the T1‐MPRAGE sequence was implemented with inline AlignedSENSE reconstruction via the proposed framework. The conventional scanner reconstruction (GRAPPA) was returned first for early review (A). Intermediate sequences proceeded without interruption in scanning or reconstruction (B). After the AlignedSENSE reconstruction completed, a retrieval scan was triggered to return the AlignedSENSE‐reconstructed image to the MR console (C). Compared to the scanner‐reconstructed image, the AlignedSENSE result demonstrated equivalent image properties, with the added benefit of effective removal of motion related artifacts.

Screenshots from the MR console demonstrate successful inline integration of AlignedSENSE on a T1‐MPRAGE sequence. The native scanner‐reconstructed image was returned for early review (Figure 3A). The AlignedSENSE reconstruction (Figure 3B) proceeds on the remote computer whilst scanning continued uninterrupted, and once it was complete, the resulting images were retrieved via a retrieval scan (Figure 3C). All sequences, including intermediate scans, were scanned and reconstructed without delay, confirming the framework's non‐disruptive integration with standard clinical workflows.

**FIGURE 4:**
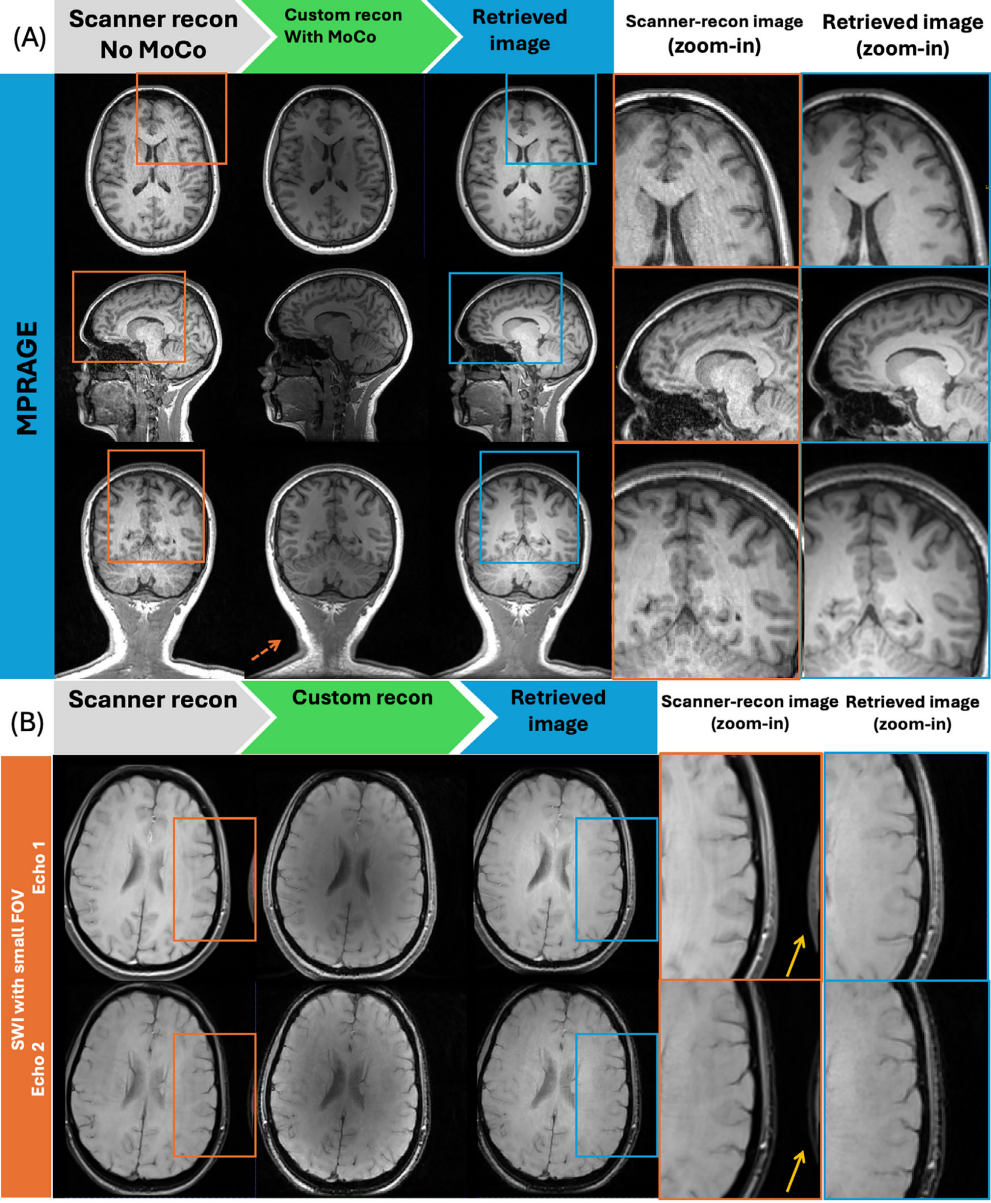
Comparison of scanner‐reconstructed, AlignedSENSE‐reconstructed images before retrieval, and AlignedSENSE‐reconstructed images after retrieval (from left to right) for the T1‐MPRAGE (A) and SWI (B) sequences. Successful application of scanner‐based bias field and distortion correction can be observed on the retrieved images (orange dashed arrows), while the AlignedSENSE reconstructed images saved in the external server but prior to retrieval lack these standard post‐processing steps, resulting in visible differences from the scanner reconstructions. Demonstrating the framework's capability to support multi‐input reconstructions, the wrapping artifact seen in the scanner‐reconstructed SWI image (yellow solid arrow) was removed by using an external reference scan with a larger FOV for coil sensitivity estimation and reconstructing to a correspondingly larger FOV.

In Figure 4, compared with scanner‐reconstructed image (left), the retrieved image (right) maintained consistency through the successfully applied scanner‐based corrections (i.e., bias field and distortion correction for our case). In contrast, these corrections were absent in the non‐retrieved image (i.e., images reconstructed and saved in the external server but prior to retrieval to MR console, middle) produced by the remote reconstruction code (note the image shading and lack of geometry correction in the neck, orange dashed arrow). Additionally, the framework's support for multiple inputs was demonstrated by eliminating a wrapping artifact by reconstructing to a larger FOV using an external reference scan for coil sensitivity estimation for SWI (Figure 4B, yellow arrow).

### 3.2 Feasibility for Multiple Inline‐Implemented Sequences Within One Examination

**FIGURE 5:**
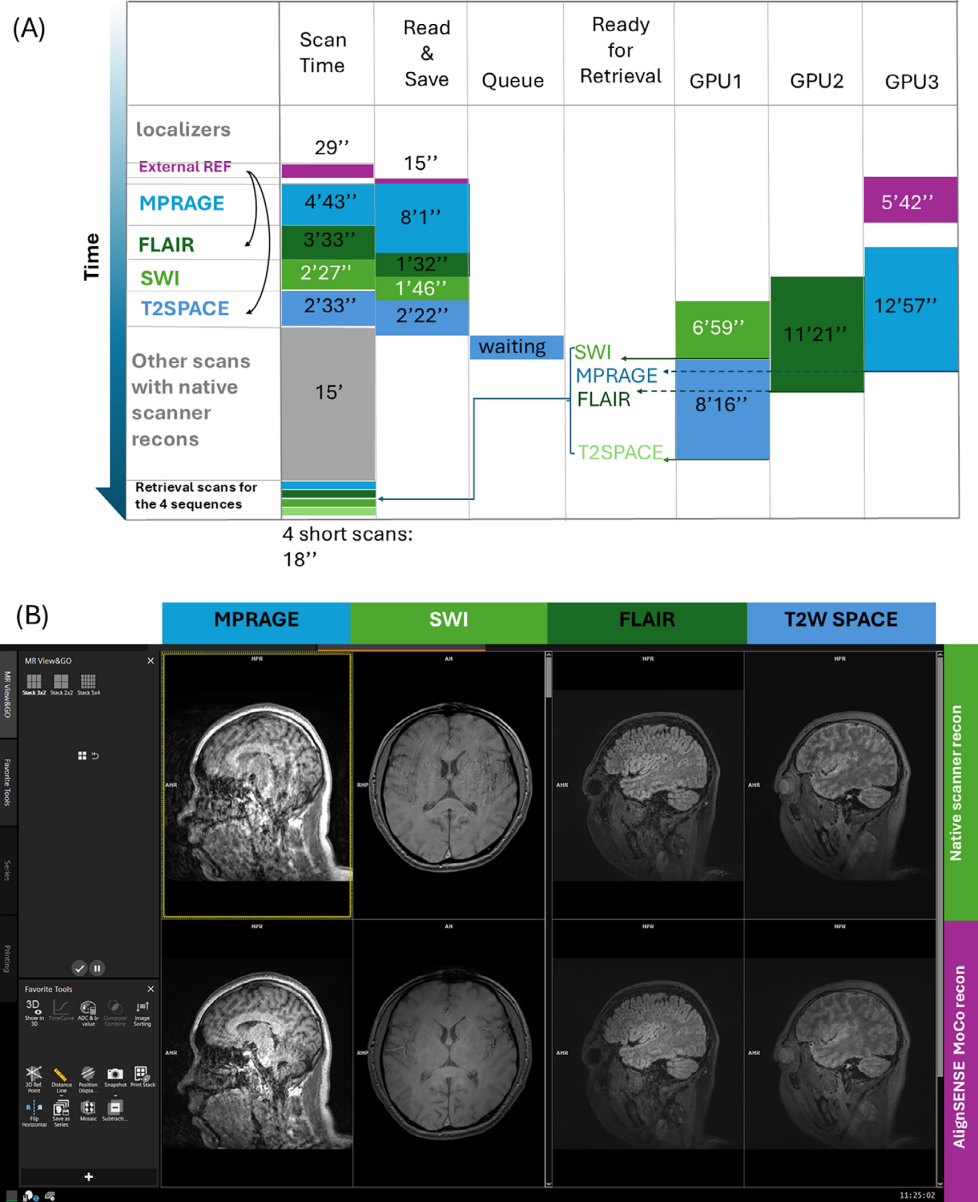
Example of resource management and ability of asynchronous reconstruction framework to enable uninterrupted scanner operation while the remote server completes its tasks. (A) Timeline diagram for the full protocol showing the four sequences implemented inline AlignedSENSE reconstruction for motion correction and the external reference for coil sensitivity estimation, and (B) screenshots on the MR console with scanner‐reconstructed images of the four sequences in the top row and inline AlignedSENSE‐reconstructed images in the bottom row. Data from the first three sequences was reconstructed in parallel on three GPUs, while data from the fourth sequence was automatically queued. After the first sequence was completed, the fourth sequence reconstruction was triggered in parallel. Finally, the reconstructed images saved on the server were retrieved to the MR console via retrieval scans.

The neuroimaging protocol with five sequences implemented inline via the proposed framework was successfully executed without disrupting scanning or reconstruction. From the timeline (Figure 5A), all sequences ran sequentially without delay. Standard scanner reconstructions provided immediate console review (Figure 5B, top row). Motion‐corrected reconstructions (“Moco recon”) for the first three sequences were distributed across three GPUs and processed in parallel. The final sequence was queued and automatically launched once resources became available, demonstrating the framework's resource‐aware scheduling. Finally, all custom‐reconstructed images were successfully retrieved using retrieval scans (Figure 5B, button row).

### 3.3 Robustness

Between April 3, 2024 and April 4, 2025, 480 subjects were scanned for the TwinsUK Project using a neuroimaging protocol that included T1‐MPRAGE with inline motion correction (AlignedSENSE) reconstruction. No disruptions or delays were reported. A retrospective server database review showed that motion‐corrected images were successfully generated for all 480 cases. A retrospective PACS review showed that 476 cases (99%) included both scanner and motion‐corrected images. Of these, 456 cases (95%) were retrieved during the examination via a retrieval scan, and 21 cases (4%) were retrieved using retro‐reconstruction afterward. In four cases (< 1%), only scanner‐reconstructed images were available in PACS.

For those four cases, reconstruction on the external server was completed, but the retrieval scans were triggered before images were ready due to extended reconstruction times (e.g., severe motion). Although retro‐reconstruction could have recovered the images, this was not done at the time and the original raw data was not retained, so this pathway closed. Since images were reconstructed on the remote server, use of a retrieval scan remained feasible, but without scanner‐based post‐processing. These cases highlight the need to choose between retrieval scans and retro‐reconstruction according to their characteristics, which are discussed in Supporting Information A.

## 4 Discussion

In this work, we have presented an open‐source framework for inline deployment of established offline MR reconstructions using Gadgetron implemented on Siemens scanners. The framework simplifies integration by addressing key practical barriers through targeted design features: data format incompatibility is resolved enabling maximum script reuse; multi‐input and long reconstruction are supported by enabling an asynchronous trigger‐and‐retrieval mechanism, resource‐aware scheduling, and integrated data management. We validated feasibility and robustness through representative reconstructions and a large‐scale clinical study, with all components and demonstration examples openly released.

While the framework addresses key barriers to inline integration, several limitations remain, so it is still under active development.

First, the framework currently depends on a full Gadgetron installation. While IceGadgetron shares substantial conceptual similarity with Siemens proprietary platforms such as FIRE and Open Recon, and the framework could in principle be integrated with these platforms with minor modifications, the current open‐source scripts and tutorials is specific to implementations based on IceGadgetron. To ease setup, detailed instructions for Gadgetron environment deployment are provided in the user manual [17]. For platforms from other vendors that lack core IceGadgetron inline data‐streaming features (i.e., the Emitter and Injector functors), the framework's logic may still offer reference value, although the provided scripts might not be directly applicable.

Second, the input converter generates a Twix‐like structure, limiting the framework's use to Siemens platforms, although other components are vendor‐independent. Additionally, the current Twix‐like data structure covers most common cases, but the ParameterMap does not yet capture all headers from the native Twix format, which may affect scripts relying on less common header fields. For example, geometry‐related metadata have not yet been fully mapped, and dedicated utility functions are currently provided to generate alternative parameters. This gap is expected to be closed in future updates. Earlier versions of the framework were tested on scanners running Siemens XA30 and VE12, confirming general compatibility, while the open‐sourced version was only validated on XA60. Given header variations between software generations, adjustments to the converter may be required for full compatibility. Clear instructions for extending the ParameterMap are provided within the manual [17]. Community contributions are invited to improve generalizability of the framework.

Third, while a complied language (e.g., C++) implementation could greatly accelerate long reconstructions and potentially eliminate the need for asynchronous trigger‐and‐retrieval operation in some cases, it requires substantial engineering effort in redevelopment and validation. The proposed framework offers a practical interim solution for inline deployment and testing of high‐level or prototype reconstructions without major code changes. If compiled reconstruction code still benefits from asynchronous deployment, the framework can easily be modified to trigger it by a direct launch bash script. Practically, the framework is currently implemented in MATLAB, so that reconstruction programs developed in other programming languages or as standalone executables need to be embedded within MATLAB scripts for compatibility, which is not always straightforward. A Python‐based version is in development to broaden accessibility, for which the output data structure of twixtools [27] is being considered in the design of the general input converter of this version. Conversely, for sequences requiring short and real‐time reconstruction, the original Gadgetron platform already provides adequate support, and such use cases therefore fall outside the scope of the asynchronous design proposed here. Nonetheless, we hope that the tools introduced in this work, such as the general input converter, may still help to simplify implementation in these scenarios.

Fourth, resource monitoring currently focuses on GPU availability, whereas the same logic can be extended to monitor CPU and RAM usage.

Fifth, image retrieval must occur after custom reconstructions are complete; otherwise, no images are returned, as seen in the four failed retrieval cases in the Twins Project. Several approaches could address this issue. One option is that retrieval scans can be repeated during the examination, and failed attempts do not disrupt subsequent scans or reconstructions, while retro‐reconstruction after the session offers a reliable fallback when the subject has left the table. Another potential alternative is Siemens' Prio Recon feature, which establishes an independent reconstruction pathway parallel to the main one and can potentially automate image returns of long custom‐reconstructions without separate retrieval and disruption of reconstructions in the main pathway. However, as both pathways share system resources and are sequential, it restricts large 3D sequences and multiple (> 2) concurrent reconstructions. A potential future enhancement is a console‐side notification triggered immediately upon reconstruction completion, improving robustness and user experience.

Looking ahead, as interest in open platforms and inline reconstruction grows, issues such as data format incompatibilities may recede if standardized interchange specifications (e.g., ISMRMRD) become the norm for both offline and inline deployment. It remains to be seen whether other frameworks will offer the same flexibility for multi‐input and asynchronous reconstruction with simple retrieval pathways to the scanner console and database, enabling inline deployment without affecting examination structure or operation.

In summary, the proposed framework lowers the technical barrier for inline deployment of established offline reconstructions with minimum modification, regardless of input numbers or reconstruction time. It was validated as suitable for both routine clinical use and large‐scale cohort studies.

## Funding

The authors acknowledge financial support from: the Wellcome Trust, Medical Research Council, Versus Arthritis, European Union Horizon 2020, Chronic Disease Research Foundation (CDRF), Wellcome Leap Dynamic Resilience Programme (co‐funded by Temasek Trust), Zoe Ltd. This research was supported by the National Institute for Health and Care Research (NIHR) Clincial Research Facility (CRF) and HealthTech Research Centre in Cardiovascular and Respiratory Medicine (HRC) at Guy's and St Thomas' NHS Foundation Trust. The views expressed are those of the author(s) and not necessarily those of the NHS, the NIHR or the Department of Health and Social Care. L.C.‐G. acknowledges funding from Project PID2024‐162095OB‐I00, funded by MICIU/AEI/10.13039/501100011033 and co‐financed by FEDER, UE.

## Conflicts of Interest

Sarah McElroy is an employee of Siemens Healthcare Limited. The other authors declare no conflicts of interest.

## Supporting information


**Figure S1.** Screenshot of the MR console interface showing the drop‐down menu (“Gadget Mode”) for selecting the ICE configuration file. The option “DISORDER Gadg” is used for the custom AlignedSENSE reconstruction, while “Pull DISORDER highres” is used for image retrieval.
**Figure S2.** The diagram for the matching mechanism of locating the image file to be retrieved for a specific retrieval scan or retro‐reconstruction.
**Table S1.** The inline deployment details of SENSE, AlignedSENSE, and NUFFT reconstructions.
**Table S2.** The sequence parameters and reconstruction information of the demonstration sequences for inline implementation.
**Table S3.** The sequence parameters and reconstruction information of the protocol.

## Data Availability

The data that support the findings of this study are openly available in Practical_Inline_Recon_Framework‐public at https://github.com/ZihanNing/Practical_Inline_Recon_Framework‐public.git.
