# Supplementary material for: From Offline to Inline Without Pain: A Practical Framework for Translating Offline MR Reconstructions to Inline Deployment Using the Gadgetron Platform
Source: Magn Reson Med. 2026 Apr 1;96(1):448–59. doi: 10.1002/mrm.70304 (PMC13156422; doi:10.1002/mrm.70304)
Supplement: Supplementary file 1 — Figure S1. Screenshot of the MR console interface showing the drop‐down menu (“Gadget Mode”) for selecting the ICE configuration file. The option “DISORDER Gadg” is used for the custom AlignedSENSE reconstruction, while “Pull DISORDER highres” is used for image retrieval. Figure S2. The diagram for the matching mechanism of locating the image file to be retrieved for a specific retrieval scan or retro‐reconstruction. Table S1. The inline deployment details of SENSE, AlignedSENSE, and NUFFT reconstructions. Table S2. The sequence parameters and reconstruction information of the demonstration sequences for inline implementation. Table S3. The sequence parameters and reconstruction information of the protocol. [file MRM-96-448-s001.docx]

## Section A: Retrieval Scans and Retro-reconstruction

Two strategies are available for returning reconstructed images to the console: (A) a retrieval (fast dummy) scan that can be predefined in the protocol and executed automatically, and (B) a retro-reconstruction that is manually configured and triggered after the examination. Successful image retrieval requires four conditions to be met: (1) the attached ICE configuration files must specify the image-retrieval pipeline; (2) the parameters related to matrix size of the retrieval scan or retro-reconstruction must align with those of the target image; (3) their sequence names must follow the defined matching rule to associate the desired image to be retrieval; and (4) the retrieval must be triggered at an appropriate time.

(1) ICE configuration files

The ICE configuration files (.xml files) define configurations related to IceGadgetron program, including which reconstruction pipe to execute, where to insert the injector and emitter functors, etc. To enable image retrieval, the retrieval sequence or retro-reconstruction must switch its ICE configuration from the custom reconstruction pipeline to the image retrieval pipeline. In our implementation, for retrieval scans, a drop-down “Gadget Mode” menu is available in the MR console interface for selecting the ICE configuration (Fig. S1); while for retro-reconstructions, the configuration can be modified manually on the console. A step-by-step instruction and ICE configuration templates are provided in the open-source repository.


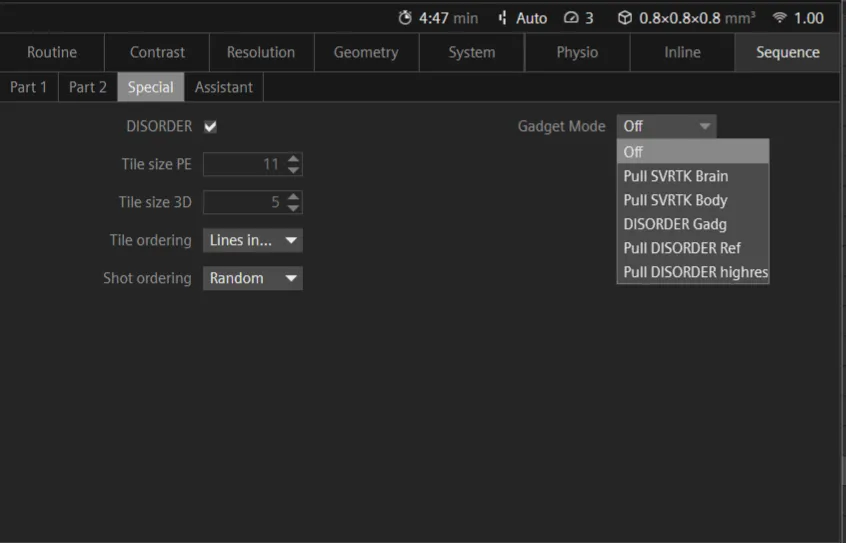


Figure S1. Screenshot of the MR console interface showing the drop-down menu (“Gadget Mode”) for selecting the ICE configuration file. The option “DISORDER Gadg” is used for the custom AlignedSENSE reconstruction, while “Pull DISORDER highres” is used for image retrieval.

(2) Matrix size alignment

Because of the characteristics of the Siemens reconstruction pipeline, only images whose matrix size matches that of the retrieval scan or retro-reconstruction can be accepted by the console. This is inherently ensured for retro-reconstruction, but for retrieval scans it is essential to align all matrix-related parameters with those of the target acquisition. The simplest way to achieve this is to duplicate the target scan and switch its attached ICE configuration to the image-retrieval pipeline, thus guaranteeing matrix compatibility. To minimise retrieval time, the duplicated sequence can be shortened by adjusting parameters such as acceleration rate, removing preparation pulses, or reducing TR, while keeping the same matrix size. However, this modification changes the DICOM header of the retrieved image, as the header reflects the parameters of the retrieval sequence instead of the target sequence. To avoid such discrepancies, we implemented retrieval sequences specifically designed for each target sequence: when the ICE configuration pipeline is set to “retrieval”, the sequence acquires only a single central k-space line but retains all header information identical to the target scan.

To further prevent retrieval failure due to mismatched matrix sizes, a matrix-check module in the retrieval script automatically detects discrepancies and interpolates or crops the image if needed, with a warning logged on the server. We nonetheless recommend avoiding such corrections where possible. For fixed research protocols, as in most cohort studies, matrix alignment is easily maintained. If scan parameters (e.g., FOV) are modified during examination, the retrieval sequence must be updated accordingly, or retro-reconstruction should be used instead.

In addition, owing to the matrix alignment requirement and the mechanism of the Siemens reconstruction pipeline, where each scan or retro-reconstructions is expected to generate its own reconstructed images, each target scan currently requires its own retrieval scan or retro-reconstruction. A single retrieval sequence or retro-reconstruction cannot return multiple images with differing matrix dimensions.

(3) Matching rules

For each subject scanned on a given day, the framework creates a folder on the external server named ‘ScanDate_SubjectID’, which stores all raw data and reconstruction outputs for that subject on that day. After a custom reconstruction completes, two copies of the image prepared for retrieval are saved in this folder. The first copy includes the retrieval label, the target scan name and a timestamp to prevent overwriting (e.g., image_to_be_retrieved_TargetScanName_timestamp); while the second copy includes the retrieval label and the sequence type of the target scan (e.g., image_to_be_retrieved_TargetScanSequenceType) and may be overwritten if the same sequence type is acquired multiple times during the same examination.

To allow a retrieval scan or retro-reconstruction to locate the correct file, the provided “image retrieval” ICE pipeline uses a matching mechanism illustrated in Fig. S2. In principle, two conditions must be satisfied.

First, the retrieval must occur on the same scan day and under the same subject ID as the target scan, ensuring that the correct folder is accessed. If the retrieval cannot be triggered on the same day, the current matching logic will default to the folder corresponding to the most recent scan date for that subject ID.

Second, apart from the suffix “_retrieval” or “_dummy”, the retrieval scan or retro-reconstruction should duplicate the target scan name. This requirement is inherently fulfilled for retro-reconstruction. However, for retrieval scans, the sequence name should be set accordingly. Although several fallback rules are included in the matching mechanism (Fig. S2), we recommend following this naming rule to ensure correct image retrieval.

For the occasions that none of the matching conditions could be met, a “debug mode” is available as a final fallback. In this mode, users may place breakpoints in the MATLAB program running inside the framework and manually select the file to be retrieved. Detailed guidance is provided in Section 1.3 of the user manual in the open-source repository.

(4) Retrieval timing

Because one of the motivations for the framework design is to support long custom reconstructions, image retrieval must be triggered only after the reconstruction has completed. Reconstruction progress can be monitored in real time through the logs generated on the external server, and any retrieval triggered prematurely will return no image. Approaches to mitigate this issue are described in the Discussion section of the main text (fifth point).


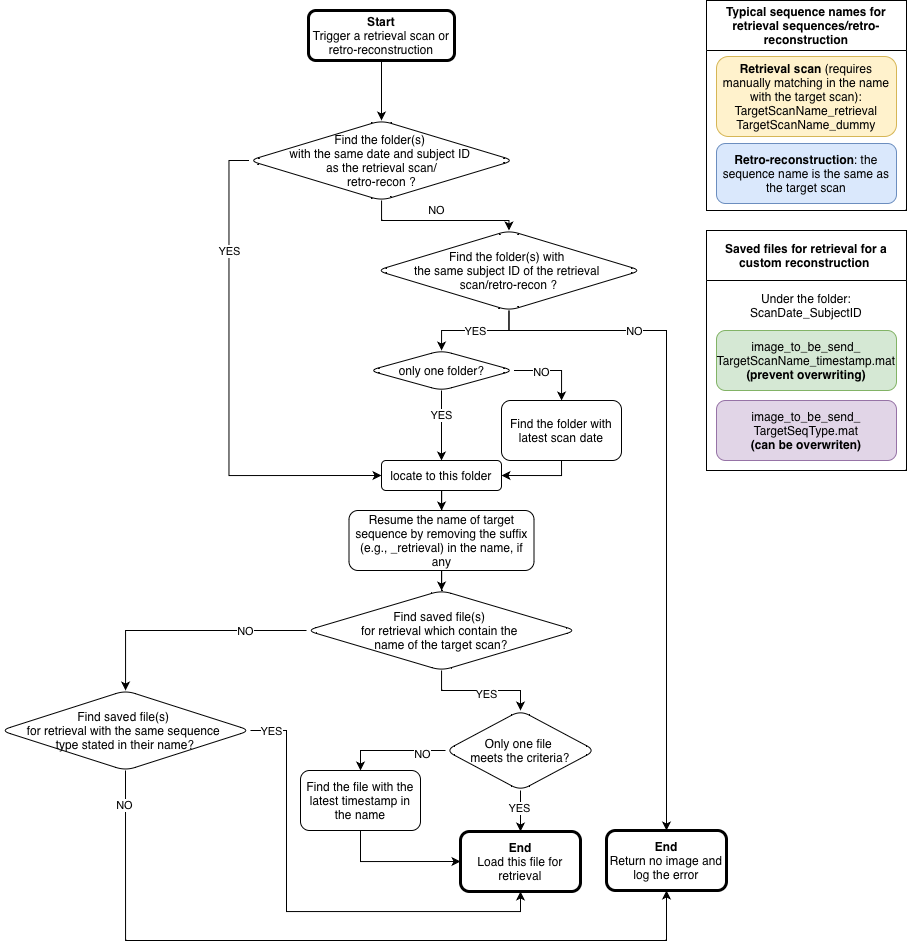


Figure S2 The diagram for the matching mechanism of locating the image file to be retrieved for a specific retrieval scan or retro-reconstruction.

Table S1. The inline deployment details of SENSE, AlignedSENSE, and NUFFT reconstructions

| Reconstructions deployed inline | MR scanner and sequences | External server |
| --- | --- | --- |
| SENSE ^12^ and AlignedSENSE ^13,14^ reconstructions | 3T MR scanner (MAGNETOM Vida, Siemens Healthineers, Forchheim, Germany) running XA60 software, using SWI and MPRAGE sequences | Intel® Xeon® Silver 4314 CPU  125 GB RAM, 32 GB swap memory  Three NVIDIA GeForce RTX A6000 GPUs (48 GB memory each) |
| NUFFT reconstruction^18^ for ^23^Na imaging^19,20^ | 7T MR scanner (MAGNETOM Terra.X, Siemens Healthineers, Forchheim, Germany) running XA60 software with a multi-echo GRE sequence with radial trajectory | Intel® Core™ i9-10920X CPU  125 GB RAM, 64 GB swap memory  Two NVIDIA GeForce RTX 2080 Ti GPUs (11 GB memory each) |

Table S2. The sequence parameters and reconstruction information of the demonstration sequences for inline implementation

|  | External reference | SWI | T1-MPRAGE | Radial multi-echo GRE |
| --- | --- | --- | --- | --- |
| Field strength | 3 T | 3 T | 3 T | 7 T |
| Field of view, mm^3^ | 256×256×288 | 230×204×144 | 256×256×208 | 200×200×200 |
| Resolution, mm^3^ | 4×4×4 | 0.8×0.8×3 | 1×1×1 | 3.1×3.1×3.1 |
| TE(/ΔTE)/TR, ms | 1.55/417 | 9.42/10.28/27  (2 echoes) | 1.97/2000 | 0.35/4/151  (38 echoes) |
| TI, ms | N.A. | N.A. | 880 | N.A. |
| Flip angle, deg | 4 | 15 | 8 | 90 |
| Acceleration | None | GRAPPA 2×1 | GRAPPA 2×1 | None |
| Partial Fourier | N.A. | Phase: 7/8  Slice: 7/8 | N.A. | N.A. |
| Turbo factor | 72 | N.A. | 208 | N.A. |
| DISORDER related^14^ | N.A. | N.A. | Tile size, 15×9 Random-checkered | N.A. |
| Scan time | 29s | 2min 59s | 4min 43s | 15min 06s |
| Custom reconstruction | ESPIRiT for coil sensitivity estimation^16^ | SENSE reconstruction^12^ | AlignedSENSE reconstruction^13,14^ | NUFFT^18,19,20^ |
| Reconstruction time* | ~6min | ~12min | ~13min | ~3min |

* To note, the reconstruction time depends on the size and complexity of the raw data (e.g., motion severity) as well as the available computational resources.

Table S3. The sequence parameters and reconstruction information of the protocol

|  | SWI  (smaller FOV) | FLAIR | T2 SPACE |
| --- | --- | --- | --- |
| Field of view, mm^3^ | 180×160×144 | 250×250×160 | 250×250×160 |
| Resolution, mm^3^ | 0.8×0.8×3 | 1×1×1 | 1×1×1 |
| Imaging view | Transverse | Sagittal | Sagittal |
| TE(/ΔTE)/TR, ms | 9.42/10.28/27  (2 echoes) | 394/8000 | 408/3200 |
| TI, ms | N.A. | 2300 | N.A. |
| T2 Prep duration, ms | N.A. | 125 | N.A. |
| Flip angle, deg | 15 | T2 Variable Scheme | T2 Variable Scheme |
| Acceleration | GRAPPA 2×1 | CAIPIRINHA 2×2 | CAIPIRINHA 2×2 |
| Partial Fourier | Phase: 7/8  Slice: 7/8 | N.A. | N.A. |
| Turbo factor | N.A. | 278 | 278 |
| DISORDER related^14^ | Tile size, 5×8  Random-checkered | Tile size, 8×3  Random-checkered | Tile size, 8×3  Random-checkered |
| Scan time | 2min 8s | 3min 33s | 1min 33s |
| Custom reconstruction | AlignedSENSE reconstruction^13,14^ | | |
| Reconstruction time^†^ | ~7min | ~12min | ~8min |

* The sequence parameters and reconstruction information of the external reference and T1-MPRAGE sequences were the same as shown in Table S2

^†^ The reconstruction time depends on the size and complexity of the raw data (e.g., motion severity) as well as the available computational resources.
